# The Attitude of Great Writers Towards Disease

**Published:** 1892-10-15

**Authors:** 


### The Attitude of Great Writers Towards Disease.

### XIII.—ELIZABETH BARRETT BROWNING.

For those who, like many of our readers, are brought into close daily contact with suffering "in mind, body, and estate" of an acuteness they dare not attempt to realise, a special value must lie in the ripest thoughts about pain of her who looked back from a golden autumn to tell the world what God had taught her in the season of storm and desolation. It is significant that the work in which Mrs. Browning set herself to grapple with the terrible problems connected with suffering and disease, was produced not in the darkened room where the stern lesson had been learned, but in the sunny City of Flowers when long dormant joys were blossoming under the love of husband and child. There had never been a time when she had not striven to understand the uses of pain. "If knowledge is power, suffering should be acceptable as a part of knowledge." So she wrote in the preface to one of her earlier poems, applying to herself the poet's words,

We learn in suffering what we teach in song.

And in her beautiful plea for the outcast children of London she puts this thought foremost (not always remembered in this practical age) in the purpose of the schools it was proposed to build:—

Where the outcasts may to-morrow
Learn by gentle words and rules
*Just the uses of their sorrow.*

But the pain which lies too close to the heart must always defy analysis. While the yoke is round the neck the form and substance of the burden may be guessed at, but never fully understood. Aurora Leigh could hardly have been produced but for the health and happiness which later in the life of the authoress placed her in a different point of view to sorrow, and rounded her experience with the realisation of all a poet's dreams.

Aurora Leigh is still, in spite of much adverse criticism, a power among us. It is no small tribute to England's greatest poetess that a recent writer in the *Revue des Deux Mondes*, after an appreciative analysis of the poem (lately translated into French), hails it as the forerunner of much that is noblest in the current of modern thought.

It is impossible not to recognise in it a very considerable autobiographical element. Though it presents the inner life and experience of two persons, it is in reality the story of the development of a soul, viewed under two aspects. In the method only by which the aim is to be worked out do they diverge, and the poem is intended to show the maimed life of each, cut asunder from its proper complement.

Natural things
And spiritual—who separates those two
In art, in morals, or the social drift,
Tears up the bond of nature and brings death. . . .
Leads vulgar days, deals ignorantly with men,
Is wrong in short at all points.

Here we find typified the twofold aspect of the poet's life: one half bent to earth, spending itself for men, burnt up with passionate hatred for the forms of evil which injure the weak; the other half lifted up to heaven, dwelling in a purer atmosphere, under the immediate eye of God. One half is represented by Romney, the other by Aurora, but both are reflections of the inner life of Elizabeth Barrett.

Now the blending into one ideal being is the work of suffering. Here is the great Master who is to teach Aurora love and Romney faith, and finally unite them

As sword that, after battle, flings to sheath.

Romney's is the re-awakened enthusiasm for humanity of the age, and he gives himself early to the cause of Man as distinct from men.

Dear, my soul is gray
With poring over the long sum of ill.
May I choose indeed
But vow away my years, my means, my aims
Among the helpers, if there's any help
In such a social strait?

And when Aurora cannot follow him nor surrender her devotion to art for a combat with evils of which she knows nothing, he taunts her with a woman's limited sympathies.

You weep for what you know. A red-haired child
Sick in a fever, if you touch him once,
Though but so little as with a finger tip
Will set you weeping; but a million sick—
You could as soon weep for the rule of three
Or compound fractions.

Aurora, mistrusting his methods, holds firm her purpose to dedicate herself to Art and God.

It takes a soul
To move a body; it takes a high-souled man
To move the masses even to a cleaner stye;
It takes the ideal to blow a hair's breadth off
The dust of the actual.

And so begins the weary struggle of each in the domain which they have chosen. Romney finds his reforms a series of blunders, and his aims universally misunderstood. His greatest blunder lies in his dealings with Marian Erle, the daughter of the people, of whom he proposes to make a help-meet in his work. A very beautiful creation is Marian; an embodiment of the belief that no outward degradation can affect a pure soul, but that it rather serves to refine, and glorify in proportion to the greatness of the trial.

You believe
In God for your part? Ay? That He who makes
Can make good things from ill thing, best from worst,
As men plant tulips upon dunghills when
They wish them finest?

Romney, with all his thoughts bent earthwards, and his hopes stopping short at relief of present suffering, can do little for such a soul. In the end he owns

Heaven's angels round her life suffice
To fight the rats of our society
Without this Romney.

Then comes the final overthrow of all his schemes, when his life is attempted and his sight destroyed by those to whose cause he had surrendered himself. And at length, in blindness and failure, in the actual experience of the evil he had been trying to lift from others, he discovers light and hope undreamed of before. He tastes misery, and straightway rises above it.

From his personal loss
He has come to hope for others when they lose,
And wear a gladder faith in what we gain.
Through bitter experience, compensation sweet.

For there breaks upon him in the great isolation of his blindness a revelation that

The man most man, with tenderest human hands,
Works best for men—as God in Nazareth.

In the consciousness of his own weakness he finds true peace, and learns to stand

And work among Christ's little ones, content,
Raising men's bodies still by raising souls.

To Aurora the sense of failure is equally present in the moment of her highest apparent success as a poetess. The individual sense of responsibility and of sympathy with her kind comes to her at length by the same road of pain. The pain of an unrequited love, the bitter knowledge of having helped to blight the life dearest to her, the long loneliness and struggle for artistic perfection, remote from ties of love or kinship, awaken her at length to a perception of what she has always lacked.

Passioned to exalt
The artist's instinct in me at the cost
Of putting down the woman's, I forgot
No perfect artist is developed here
From any imperfect woman . . .
. . . Art is much, but love is more.

Thus in Mrs. Browning's "highest convictions upon life and art" we find pain is, after all, the keystone of the arch, uniting and perfecting the whole. To all who strive as they strove to lessen the great sum of human misery, and, striving, feel their impotence and daily learn to bow before a Power mightier than any human science of healing or reform, she brings a message, never more needed than now, of wise warning and encouragement:

Though we fail indeed
You—I—a score of such weak workers, . . He
Fails never. If He cannot work by us,
He will work over us. Does he want a man,
Much less a woman, think you? Every time
The star winks there so many souls are born,
Who all shall work too. Let our own be calm.

